# Proceedings from the 2^nd^ Next Gen Therapies for Systemic Juvenile Idiopathic Arthritis and Macrophage Activation Syndrome symposium held on October 3-4, 2019

**DOI:** 10.1186/s12969-020-00444-7

**Published:** 2020-07-15

**Authors:** Scott W. Canna, Grant S. Schulert, Adriana de Jesus, Alex Pickering, Hermine Brunner, Massimo Gadina, Stewart Levine, Raphaela Goldbach-Mansky, Jonathan Boutelle, Rashmi Sinha, Fabrizio DeBenedetti, Alexei Grom, on behalf of the NextGen 2019 Participants, Beth Gottlieb, Rae Yeung, Mona Riskalla, Sampath Prahalad, Sara Alehashemi, Shan Chandrakasan, Bas Vastert, Yuki Kimura, Anna Carlson, Emily Schumacher, Regina Minerva, Jonathan Pierce, Kate Pierce, Zulayka Martinez, Kari Cupp, Leah Bush, Wendy Costello, Vincent DelGaizo

**Affiliations:** 1 University of Pittsburgh, Pittsburg, PA USA; 2 Division of Rheumatology, Cincinnati Children’s Hospital Medical Center and Department of Pediatrics, University of Cincinnati College of Medicine, Cincinnati, USA; 3 Intramural Research Program, National Institute of Allergy and Infectious Diseases, NIH, North Bethesda, USA; 4 Harvard Medical School, Boston, MA USA; 5 SJIA Foundation, Cincinnati, USA; 6 Ospedale Pediatrico Bambino Gesù, Rome, Italy

**Keywords:** Systemic juvenile idiopathic arthritis, Macrophage activation syndrome, Hemophagocytic lymphohistiocytosis, Interferonopathy, Interstitial lung disease, Pulmonary alveolar proteinosis, Alveolar macrophages

## Abstract

For reasons poorly understood, and despite the availability of biological medications blocking IL-1 and IL-6 that have markedly improved overall disease control, children with Systemic Juvenile Idiopathic Arthritis (SJIA) are now increasingly diagnosed with life-threatening chronic complications, including hepatitis and lung disease (SJIA-LD). On October 3–4, 2019, a two-day meeting, *NextGen Therapies for Systemic Juvenile Idiopathic Arthritis (SJIA) & macrophage activation syndrome (MAS*) organized by the *Systemic JIA Foundation* (www.systemicjia.org/) in Washington, DC brought together scientists, clinicians, parents and FDA representatives with the objectives (1) to integrate clinical and research findings in MAS and SJIA-LD, and (2) to develop a shared understanding of this seemingly new pulmonary complication of SJIA. The current manuscript summarizes discussions and conclusions of the meeting.

## Introduction

Systemic Juvenile Idiopathic Arthritis (SJIA), formerly Still’s Disease, is an autoinflammatory disorder known for marked systemic, predominantly innate immune activation [1]. The adult counterpart is Adult Onset Still’s Disease [2]. About 15% of SJIA patients will also develop overt macrophage activation syndrome (MAS), an episodic hyper-inflammatory state caused by excessive activation and expansion of macrophages with hemophagocytic properties and predominantly CD8+ T lymphocytes [3]. It is accompanied by a surge in IFN-γ and downstream chemokines [4], as well as strikingly high serum levels of IL-18 [5]. MAS has been associated with significant morbidity and mortality. For reasons poorly understood, and despite the availability of biological medications blocking IL-1 and IL-6 that have markedly improved overall disease control [6, 7], MAS rates have remained unchanged [8–10]. Additionally, children with SJIA and MAS are now increasingly diagnosed with life-threatening chronic complications, including hepatitis and lung disease (SJIA-LD) [11–13]. Evaluation of lung biopsies of these patients reveals severe inflammation with features of pulmonary alveolar proteinosis (PAP), endogenous lipoid pneumonia, and fibrosis [12, 13], all supporting that SJIA_−_LD is chronic and likely diagnosed late when irreversible tissue damage has already occurred. Hence, there is a profound scientific gap of how to diagnose SJIA-LD early, elucidate risk factors, and develop therapeutic strategies to improve its prognosis.

### Conference goals and overview

As MAS and lung disease (LD) in SJIA appear to be related, on October 3–4, 2019, a two-day meeting, *NextGen Therapies for SJIA & MAS* organized by the *Systemic JIA Foundation* (www.systemicjia.org/) in Washington, DC brought together scientists, clinicians, parents and FDA representatives with the objectives (1) to integrate clinical and research findings in MAS and SJIA-LD, and (2) to develop a shared understanding of this seemingly new pulmonary complication of SJIA. In her introduction, Dr. Sinha, the President of the *Systemic JIA Foundation*, stated that the longer-term goals of the meeting were to develop a strategy to enable the early diagnosis of SJIA-LD and improve the management of SJIA-LD, based on the current state of knowledge of this condition. The meeting included in depth presentations and open discussions of unpublished research findings. Dr. Sinha emphasized the role of parental participation in this process, to ensure that their voices are heard in planning of future observational studies and clinical trials.

#### Section 1: patient perspectives

### Understanding the patient experience of SJIA-LD / moderator: Dr. Rashmi Sinha; panelists: Anna Carlson, Regina Minerva, Emily Schumacher

Three parents of children diagnosed with SJIA-LD reported their child’s experience, highlighting the lack of apparent pulmonary symptoms prior to diagnosis of SJIA-LD, a common feature that may contribute to delay in diagnosis. These testimonials provided crucial detail and context for the natural history of SJIA-LD, and are consistent with the pictures offered by three recent publications [12–14]. All three patients have had a rather resistant systemic component of the disease with recurrent MAS, and as a result, have been exposed to multiple medications including several biologics. The parents emphasized the need for relatively safe therapies that could be initiated preventively when a child shows early signs of MAS and/or the lung disease, instead of the highly immunosuppressive treatment regimens that are typically used for full-blown MAS.

### Patient story - Anna Carlson

Ms. Carlson’s daughter was diagnosed with SJIA in November 2016, at the age of 1 year. She was started on corticosteroids and canakinumab. Within 2 months, her parents started noticing a dry hacking cough and “pneumonia” like symptoms that were unresponsive to antibiotics. Her fevers returned and canakinumab dose was increased. In May 2017, a high-resolution chest computer tomography (HRCT) was obtained and the child was diagnosed with interstitial lung disease (ILD). Overnight pulse oximetry revealed significant hypoxia (desaturation episodes down to the 40s). She was admitted to the hospital, and within a few days the lung disease progressed to respiratory failure requiring admission to an Intensive Care Unit (ICU). During this 100 day “*big sick*” (as Ms. Carlson terms this event) her child required 13 days of Extra-Corporeal Membrane Oxygenation (ECMO). After being weaned from ECMO, she developed a severe case of MAS with ferritin rapidly increasing to 280,000 ng/mL, and 10-week treatment with etoposide was initiated.

A tracheostomy tube was placed due to continuous need for ventilator and oxygen supplementation, as well as G-tube to prevent aspiration. After 2 months of being bed-ridden, her child required extensive physical, occupational and speech therapy to relearn the most basic of activities. In August 2018, about a year after “the big sick”, a lung biopsy revealed PAP. Noticeably, her child did not develop clubbing until 1 year after the start of her lung symptoms.

For the past year, Ms. Carlson’s child’s SJIA has been mostly controlled (with normal labs and no rash or fever), but the lung disease persists, and is easily exacerbated by infections, which typically prompt ICU readmissions with increasing needs for supplemental oxygen (up to 20 l per minute) and more robust ventilator support. The side effects of her immunosuppressive regimen have led to profound neutropenia, with absolute neutrophil counts falling to zero. A JAK-inhibitor was recently started, and the family is hopeful that the combination of biologics, JAK inhibitor and antibiotic prophylaxis will help her child.

Ms. Carlson emphasized the need for better medication options to control the progression of SJIA-LD, and to manage the delicate balance between adequate immunosuppression and the patient’s ability to fight infections. She is concerned that there are few available options for treating SJIA-LD, and that her daughter has tried most of those medications. She did not know what they would try next if her daughter’s disease persisted.

Following the NextGen conference, Ms. Carlson’s child has experienced new lung symptoms, such as frequent blood in sputum, significant increase in mucus production.

### Patient story - Emily Schumacher

Ms. Schumacher described the story of her daughter, who is now 6 years old. She first developed signs of SJIA-like disease in 2015 with fevers and rash, initially thought to be a viral illness. Initial treatment of her SJIA included subcutaneous injections of tocilizumab. Since there was no response, tocilizumab was replaced with high dose daily anakinra injections. Cyclosporine and methotrexate were added few weeks later. SJIA remained active, and anakinra was next replaced with canakinumab without improvement of SJIA symptoms.

Despite all the efforts to avoid corticosteroids, oral prednisolone was started in July 2016, and the child continued to remain chronically steroid dependent 3 years later. In the same year, intravenous tocilizumab was initiated with the hope that infusions would work better then subcutaneous injections. Unfortunately, the child had an unusual reaction during her 3rd infusion, with tachycardia, and tachypnea requiring additional oxygen supplementation. After this event, tocilizumab was permanently discontinued.

The family was referred for a second opinion to Cincinnati Children’s Hospital, where they were informed that the child was in the state of mild subclinical MAS. The doctors also noticed mildly increased respiratory rate and subtle clubbing of the fingers, but her HRCT showed only subtle abnormalities. A repeat HRCT 1 year later, however, suggested significantly worsening pulmonary disease; a lung biopsy performed about 1 month later confirmed PAP and SJIA-LD. At that time, mycophenolate was added to her drug regimen.

Prior to being diagnosed with SJIA-LD, Ms. Schumacher’s child never had any apparent lung symptoms, and her peripheral pulse oximetry had been consistently normal. Apart from the clubbed fingers and mildly increased respiratory rate with physical activity, the child certainly lacked overt signs of lung disease at the time of SJIA-LD diagnosis.

With the combination of SJIA, subclinical MAS, and SJIA-LD, her child has been on up to 10 daily medications including oral corticosteroids, high dose anakinra, cyclosporine, methotrexate, and abatacept. In May 2018, overt MAS prompted another hospital admission, likely triggered by cytomegalovirus (CMV). Emily was having difficulty clearing the CMV virus and required continuous antiviral treatment in addition to MAS therapy. For the last year, each attempt to decrease either corticosteroids or antivirals, was followed by early signs of subclinical MAS.

Ms. Schumacher is worried that the current cocktail of medications which seemingly cannot be weaned, is not a sustainable therapeutic strategy. She strongly feels that daily corticosteroids for long periods of time are not an appropriate treatment for children. Her child is not growing and recently developed a vertebral compression fracture. Her rheumatologist is constantly struggling to balance immunosuppression to keep the disease at bay with giving the child the ability to fight off infections.

Overall, Ms. Schumacher is concerned that options are running out to control her child’s disease. She would like better treatment choices than steroids and currently available biologics, since those fail to control her child’s disease.

After NextGen conference, Ms. Schumacher’s daughter was switched from cyclosporine to tacrolimus. That helped for a few weeks, but now again she is having frequent fevers and her laboratory test results keep raising concerns for subclinical MAS (e.g., low platelet count). Her team would like to start tofacitinib but have not been able to get insurance approval thus far.

### Patient story - Regina Minerva

Ms. Minerva’s daughter was diagnosed with SJIA in July 2017, at 13 months of age, and started on prednisolone and anakinra. As prednisolone was tapered, SJIA signs and symptoms returned with laboratory tests showing low levels of systemic inflammation. In April 2018, when her inflammatory markers rose further, she was seen by hematologist / oncologist, who ruled out malignancy, but noted that her blood cell counts were suggestive of “low grade MAS”.

**Fig. 1:**
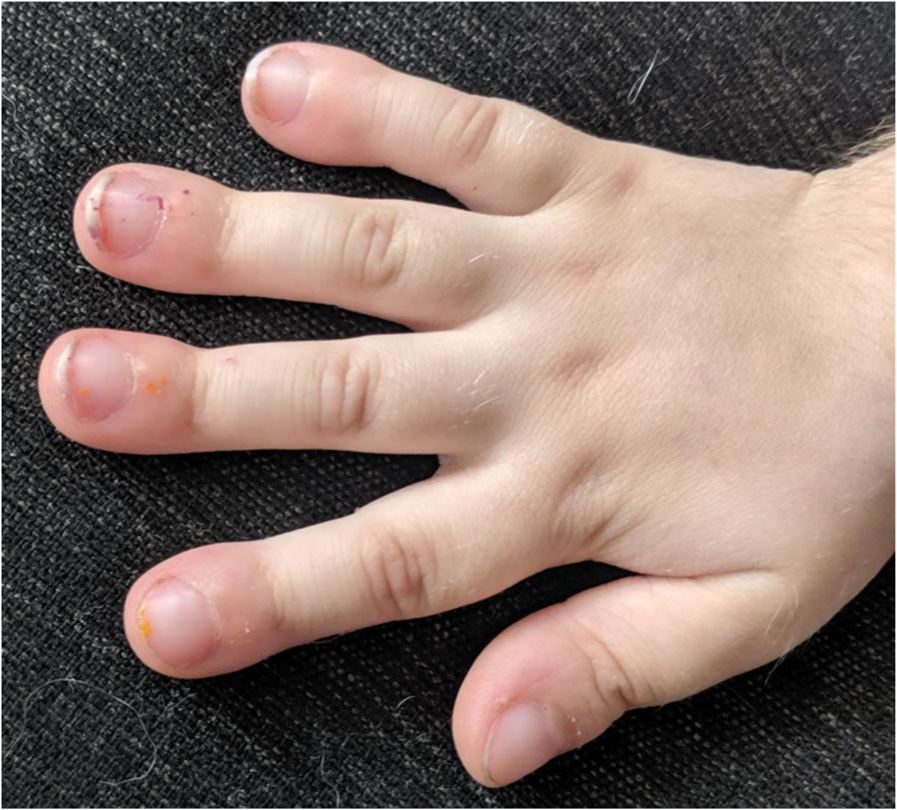
Clubbing in the fingers (*with permission of Ms. Minerva)*

In May 2018, Ms. Minerva started noticing that her child’s fingertips and toes were red and sore (Fig. 1). By September 2018, she noticed enlargement of some toes. Looking at the postings within *SJIA Parents Network*, she learnt of other children who had similar finger and toe enlargement, and were subsequently diagnosed with lung disease. In meantime, her medical team ruled out pulmonary hypertension with an ECHO. In January 2019, however her chest X-rays were abnormal. In May 2019, she was diagnosed with interstitial lung disease based on typical HRCT findings.

In May–June 2019, her child’s laboratory evaluations showed what her doctors described as “smoldering MAS”; while the trends in some symptoms and lab test results were suggestive of partial MAS, the child did not have the features of “overt MAS”. On June 22, however, the child woke up with high fever and was immediately evaluated in the emergency room where lab tests revealed full-blown MAS with ferritin of 31,000 ng/mL.

In spite of several doses of intravenous (IV) pulse corticosteroids and cyclosporine, her child’s MAS markers kept on worsening. Increasing the dose of anakinra to 10 mg/kg/day did not reverse the trend. Ms. Minerva posted about her child’s MAS in the *SJIA Parents Network*, and learned about the emapalumab clinical trial at Cincinnati Children’s. She was then connected to the doctors running the clinical trial.

In the meantime, her child’s MAS markers continued to worsen, her oxygenation started falling, and they were transferred to the ICU. Ms. Minerva and her husband requested participation in the trial. On June 28th, they were transferred to Cincinnati, and given the first dose of emapalumab as part of NCT03311854, when ferritin was at 75,000 ng/mL. After the first infusion, her fevers resolved, oxygenation returned to normal, and ferritin came down to 50,000 ng/mL. She resumed eating and drinking. By the third infusion, her ferritin came down to 7000 ng/mL, and she was returning to her normal self.

At the time of the workshop, Ms. Minerva’s child was 3 months past her emapalumab treatment. Her labs were normal with the exception of mildly increased LDH. Her breathing was normal, and the most recent HRCT showed improvement from May 2019. The girl was having the energy of a child with normal health. She remained on all the medications for SJIA, though her doctors were slowly tapering steroids. Regina was hopeful that her child would continue to do well, but she was also concerned about the return of inflammation, especially the MAS and lung symptoms. She emphasized the need for therapies that could help when a child first shows early signs of MAS and lung disease, instead of ones that can only be used when the child is in full blown MAS.

Post NextGen conference, Ms. Minerva’s child continued to feel well and had normal labs. More recently, she had a flare triggered by a viral illness with laboratory test results pointing to subclinical MAS (platelets fell, and ferritin went up to 6000 ng/mL). The dose of anakinra was increased, and a pulse corticosteroids was administered to bring the flare under control. Ms. Minerva is concerned that there are no good additional options available to keep her child’s disease under control. She believes that the only medicine that brought her child’s MAS under control, empalumab, is only available as a rescue medication (via a clinical trial), and not for ongoing or prophylactic usage.

### Discussion about patient stories

Both parents and clinicians agreed that there was a subset of children with SJIA whom clinicians should watch more closely for the development of SJIA-LD, including those with SJIA onset under age 2 years, with predominantly systemic features and limited arthritis, recurrent or “smoldering” MAS, anaphylaxis-like reaction to a biologic medication, and highly elevated serum IL-18 levels [12–14]. The parents strongly felt that for these SJIA patients, clinicians should have a low threshold for HRCT evaluation.

The group felt that as a general rule, children with SJIA under the age of 6 years who fall into this high risk group, should have a baseline chest radiograph, and overnight pulse oximetry; for the patients older than 6 years, pulmonary function testing as well as regular screening with a 6 min walk should also be performed. In the presence of any abnormalities in the above-listed tests, further imaging with HRCT of the chest is recommended. Chest CT should also be obtained if any additional features typical of SJIA-LD are present (e.g., growth failure, tachypnea, chronic non-productive cough, periungual erythema or swelling, digital clubbing as well any acute pulmonary decompensation). Episodes of routine pulmonary conditions with an abnormal chest radiograph should be followed up to ensure that the abnormal findings resolve. Parents and caregivers expressed the need for low radiation imaging options for initial and ongoing evaluation of SJIA-LD.

#### Section 2: mechanistic insights into SJIA-LD

### Report from Goldbach-Mansky & Canna labs / Dr. Raphaela Goldbach-Mansky

Dr. Goldbach-Mansky, whose recent research interests have been focused on monogenic interferonopathies, measured IFN-scores in 66 consecutive patients with no clear genetic diagnosis seen at the NIAID for presumed undifferentiated autoinflammatory diseases. Among 41 patients with elevated IFN scores, 8 had recurrent episodes of MAS, features of pulmonary alveolar proteinosis (PAP) and extremely high serum IL-18 levels, and thus, were named “*IL-18 PAP-MAS*” [14]. This subgroup of patients, whose presentation included erythematous clubbing and eventual PAP, included some described above as SJIA-LD. These 8 patients had intermediately high IFN scores, which were lower than the values observed in the monogenic interferonopathies such as CANDLE. A ratio of interferon-response genes that can be induced by IFNα/β and IFNγ over those induced by IFNα/β alone was higher in the *IL-18 PAP-MAS* patients compared to the autoinflammatory Type-I interferonopathies CANDLE and SAVI, suggesting a combined role of the Type-I IFNs (IFN-α and IFN-β) as well as Type II interferon (IFN-γ) in the immune dysregulation. Within the “*IL-18 PAP-MAS*” group, the IFN score correlated weakly with total IL-18 levels suggesting that IFN gene expression may further be co-regulated by IL-18. No unique genetic defect was identified for this group of patients, but cytokine analyses in the blood revealed elevation of hematopoietic stem cell factors that was associated with high serum IL-18 levels, a phenomenon described in other patients with high serum IL-18 levels (including the monogenic MAS predisposing disease NLRC4-MAS), but not in patients with other IL-1 mediated autoinflammatory diseases [15] or the autoinflammatory Type-I interferonopathies, CANDLE and SAVI. Elevated hematopoietic stem cell factors may indicate a distinct downstream pathway that can contribute to macrophage dysfunction in patients with high IL-18 levels [14]. The presence of free IL-18 in the bronchoalveolar lavage (BAL) fluid of patients with *IL-18 PAP-MAS* raises the question whether stem cell factors, when induced locally in the bronchial tree and or the lung, may also contribute to the alveolar macrophage dysfunction and be an additional target for treatment.

### Report from Grom & Schulert labs / Dr. Alexei Grom

Drs. Grom and Schulert reported on the Cincinnati cohort of patient with SJIA-LD that have many overlapping features with the “*IL-18 PAP-MAS*” described by Dr. Goldbach-Mansky. The vast majority of SJIA-LD patients had recurrent episodes of MAS, features of PAP and extremely high serum IL-18 levels [12]. SJIA-LD patients lacked genetic, serologic, or functional evidence of GM-CSF pathway dysfunction typical of familial or autoimmune PAP where dysfunction of alveolar macrophages leads typically to accumulation of pulmonary surfactant in alveolar space [16]. SJIA-LD lungs had a striking prominence of alveolar septal expansion by an interstitial lymphocytic infiltrate and fibrosis with remodeling that are usually absent in familial or autoimmune PAP. Additionally, in SJIA-ILD (but not familial or autoimmune PAP) the bronchoalveolar lavage (BAL) fluid contained elevated levels of IL-18 and IFN-induced chemokines CXCL-9 and -10. Transcriptional profiling of SJIA-ILD lung tissue identified upregulated T-cell and interferon activation networks, again suggesting pathophysiologic overlap with the cohort described by Dr. Goldbach-Mansky.

Due to significant overlap in genes induced by type I and type II interferons, the relative involvement of these cytokines in SJIA-LD still needs to be clarified. However, the fact that most patients with SJIA-LD have had MAS, suggests that the inflammatory cytokine milieu that drives macrophage dysfunction in MAS may also promote dysfunction of alveolar macrophages. Interferon gamma (IFN-γ) has been increasingly recognized as a pivotal cytokine in MAS [4], and therefore, the IFN-induced signature in the lung biopsies is most likely driven by IFN-γ. Consistent with this hypothesis, mice with T-cell restricted overexpression of T-bet driving IFN-γ production demonstrated both dysfunction of bone marrow macrophages, resulting in erythrophagocytosis and alveolar macrophage dysfunction with development of PAP-like lung pathology [17]. Combined, these observations suggest that targeting IFN-induced pathways might be a therapeutic option in SJIA-LD. In contrast, given the presence of alveolar septal expansion and the likely different underlying defect leading to the accumulation of pulmonary proteinosis in SJIA-LD, traditional treatments for primary PAP such as whole lung lavage may not be helpful in SJIA-LD patients.

### Alveolar macrophage dysfunction in lung disease: Dr. Stewart Levine

Since lung macrophages have a clear role in the pathogenesis of SJIA-LD, Dr. Levine presented new pulmonary insights into a range of lung disorders with macrophage dysfunction that might be informative regarding the pathobiology of SJIA-LD. So far, hereditary or autoimmune PAP have served as the main reference point for the alveolar lung disease in SJIA, so it was helpful to start considering other lung diseases which also have macrophage dysfunction. The three other diseases that he presented were Lipoid Pneumonia, Asthma, and High-fat Diet-induced Lung Disease (see Table 1).

**Table 1:** Lung Diseases Caused by Macrophage Dysfunction

Disease	Macrophage Dysfunction
Pulmonary Alveolar Proteinosis (PAP)	Surfactant accumulation in alveolar macrophages and alveoli
Lipoid Pneumonia	Lipid-laden macrophages and accumulation of intra-alveolar lipids
House Dust Mite + Virus-induced Asthma	Up-regulated apolipoprotein E (APOE) production by alveolar macrophages with resultant NLRP3 inflammasome activation and secretion of mature IL-1β
High-fat Diet-induced Lung Disease	Lipid-laden macrophages, increased lung cholesterol content, increased pro-inflammatory cytokines (interferon-γ, TNF and IL-6).

Lipoid pneumonia was identified as part of the spectrum of SJIA-LD in two recent publications [12, 13]. Lipoid pneumonia is an uncommon disease characterized by the accumulation of intra-alveolar lipids and lipid-laden macrophages [18]. The typical etiology of lipoid pneumonia is due to the exogenous inhalation or aspiration of oils or lipids into the lung, but endogenous etiologies have also been described in patients with PAP, Niemann-Pick disease (which is a lipid-storage disorder) and undifferentiated connective tissue disease, as well as a consequence of airway obstruction due to tumor.

Dr. Levine reviewed that alveolar macrophage function could be pro-inflammatory in the setting of asthma. Under normal conditions, alveolar macrophages primarily receive inhibitory signals which are anti-inflammatory [19]. However, in disease states, activating signals can override the normal inhibitory mechanisms that suppress macrophage activation. Dr. Levine’s lab has shown that this can occur in asthma where pro-inflammatory stimuli, such as house dust mite (HDM) antigen, can induce apolipoprotein E (APOE) production by alveolar macrophages [20]. When APOE levels are sufficiently high, such as when a viral infection is superimposed upon HDM exposure, APOE can function as a pro-inflammatory danger signal that activates the NLRP3 inflammasome and induces secretion of mature IL-1β by alveolar macrophages.

Dr. Levine next reviewed murine models of high-fat diet induced lung macrophage dysfunction that may have some similarities with macrophage dysfunction in SJIA-LD. When mice are fed a high-fat diet, this induces systemic inflammation and oxidative stress that causes pathological lung remodeling manifested by emphysema, sarcoid-like granulomatous inflammation, pulmonary fibrosis, and pulmonary hypertension [21]. Lungs from mice fed a high-fat diet show increased numbers of pulmonary macrophages, increased lung cholesterol content, lipid-laden macrophages, and increases in bronchoalveolar fluid cytokines (IFN-γ, TNF and IL-6).

Thus, in the appropriate setting, such as high-fat diet-induced systemic inflammation or house dust mite plus viral infection, alveolar macrophages can be converted from an anti-inflammatory cell that maintains normal pulmonary homeostasis into a pro-inflammatory cell that may promote disease pathogenesis. The concept of the alveolar macrophage as a target cell that amplifies inflammation may also be relevant for SJIA-LD.

Dr. Levine reviewed that alveolar macrophage populations in the lung are heterogeneous and can be broadly divided into two categories. Tissue-resident alveolar macrophages (TR-AMs) are the most abundant immune cell in the lung under normal conditions [22, 23]. TR-AMs represent a long-lived, self-renewing population that are derived from the fetal yolk sac and liver and function to maintain normal pulmonary homeostasis and gas exchange. In contrast, monocyte-derived alveolar macrophages (Mo-AMs) are recruited into the lung from the blood in the setting of inflammation where they have cytotoxic, antimicrobial, and pro-fibrotic functions. Thus, Mo-AMs augment inflammation, which suggests a role for these cells in SJIA-LD. Once inflammation resolves and homeostasis is restored, Mo-AMs undergo apoptosis or differentiate into cells that phenotypically resemble TR-AMs. Single cell RNA-seq experiments in a mouse model of LPS-induced inflammation have identified two distinct populations of TR-AMs and three populations of MO-AMs. Thus, single cell RNA-seq might represent a method to drill deeper into macrophage pathobiology in SJIA-LD.

#### Section 3: current approaches to management and outcomes of SJIA-LD

### Learnings from Cincinnati SJIA-LD patient cohort / Dr. Grant Schulert

Dr. Schulert provided a short update on the cohort of SJIA-LD patients evaluated at Cincinnati Children’s. They have now evaluated 27 children with SJIA-LD including 9 patients since submission of their initial manuscript [12]. Of those 27 patients, only 1 (4%) has died, which is in marked contrast to the larger, multisite cohorts [11, 13]. The reasons for this improved outcome are not known, but may include earlier diagnosis, and introduction of T-cell targeted therapies, such as mycophenolate mofetil and tacrolimus, as well as azathioprine and anti-inflammatory and prophylactic antibiotic. The treatment for SJIA-LD may also adapt some of the new approaches used in the treatment for MAS rather than SJIA, specifically treatments that can target T cell activation and interferon pathways including JAK inhibition.

Dr. Schulert also noted that their practice has been continuing anti-cytokine biologics to gain control of MAS and inflammation of active SJIA as an essential step in treatment of SJIA-LD. Controlling MAS in these patients is utterly important but often challenging. Dr. Schulert also noted there are several patients in the Cincinnati SJIA-LD cohort who have markedly improved and even resolved features of lung disease over several years. Indeed, these patients were able to achieve full control of their underlying SJIA and MAS with continued biologics along with therapies noted above.

There was also some discussion regarding possible role of biologic medications and geographic distribution of patients. Biologics are used now near universally throughout North American and Europe but SJIA-LD cases seem much more prevalent in the US, and even possible regional clusters within the US. This would be consistent with other environmental factors or triggers playing a role, which needs to be considered and investigated moving forward.

### Section 4: potentially targetable pathways and candidate therapeutics for SJIA-LD

### Review of biomarkers; IL18 as a biomarker and a target / Dr. Scott Canna

Dr. Canna presented a serum proteomics study performed in collaboration with Cincinnati and Stanford aimed at the identification of novel lung disease biomarkers that was eventually presented at American College of Rheumatology (ACR) 2019 Annual Meeting [24].

IL-18 has long been a biomarker of interest in patients with SJIA and MAS. IL-18 is uniformly and dramatically elevated in patients with active MAS. It is likewise chronically elevated, regardless of disease activity, in a few rare monogenic situations including gain-of-function mutations in NLRC4 [15], XIAP-deficiency [25], and c-terminal mutations in CDC42 regardless of disease activity [26, 27]. It is more variably elevated in active and inactive SJIA, with some patients’ levels as high as in active MAS and other patients’ level near-normal. Dr. Canna presented ongoing work related to IL-18 as a biomarker of MAS risk [5, 28–31], a host susceptibility factor for the development of MAS, and it’s peculiar elevation in SJIA-LD patients out of proportion to such patients’ degree of MAS activity. The latter point was supported by an unpublished multi-center collaborative serum proteomics study presented at the ACR 2019 Annual Meeting [24].

This study also suggested novel pathways in MAS, biomarkers of lung inflammation/damage, and potentially a role for type 2 immune responses in SJIA-LD. In addition, one SJIA-LD patient has shown a promising response to compassionate-use treatment with an IL-18 blocking strategy [32], and a clinical trial of IL-18 blockade in NLRC4 and XIAP patients is ongoing (NCT03113760). Corroborating the recent work of Schulert et al. [12], Dr. Canna’s unpublished observations suggest elevation of total and free IL-18 in BAL fluid of SJIA-LD patients, but not in various controls.

IL-18 is produced constitutively by some macrophages, but also by intestinal epithelial cells, keratinocytes, and bronchial epithelial cells. It is not known where the high IL-18 in SJIA and MAS derives from. Functionally, IL-18 is best known as an amplifier of Type 1 immune responses and specifically of IFN-γ production. The connections between IFN-γ and MAS, and potentially lung disease, were discussed by Dr. de Benedetti. While, IL-18 has been shown to amplify immune responses other than IFN-γ, including anti-bacterial (Type 17) or allergic (Type 2) responses, depending on the immune context, some of the transcriptional evidence from Schulert et al. [12] suggests that Type 1 inflammatory responses/IFN-γ may be an important part of SJIA-LD.

### An overview of selective JAK-inhibitors and relevance for SJIA-LD / Dr. Massimo Gadina

Given some pathophysiologic overlap between SJIA-LD and interferonopathies, a group of auto-inflammatory diseases where targeting JAK-STAT signaling pathways often produces a good clinical response, Dr. Gadina presented an overview of this therapeutic strategy. JAKs are phosphotransferases that bind the intracellular domains of cytokine receptors and transmit signals to activate immune responses [33]. Cytokines that signal via JAKs includes many interleukins, interferons, colony stimulating factors and hormone-like cytokines (such as erythropoietin and growth hormone). The receptors for these cytokines signal via various combinations of four JAKs (JAK1, JAK2, JAK3 and TYK2). First-generation JAK inhibitors, such as tofacitinib and baricitinib block more than one JAK, and thereby can inhibit a large number of cytokines and growth factors; these and other pan-JAK inhibitors are being investigated as therapeutic agents for a wide variety of autoimmune diseases. Based on some similarities between SJIA-LD and interferonopathies such as SAVI, JAK-inhibitors (tofacitinib) have been used in a few children with SJIA-LD, with mild improvement in some of them. More data and experience are needed to understand the efficacy and safety of this class of medications in the treatment of SJIA-LD.

Dr. Gadina also presented an overview of newer, more selective JAK-inhibitors as well as their safety and effectiveness in other diseases. Few next generation JAK inhibitors have already reached the market or are in an advanced status of development. Nonetheless, it appears that selective JAK inhibitors do not necessarily reduce the risk of infection compared with first generation JAK inhibitors. Common side effects observed include infection, anemia, neutropenia, lymphopenia and hyperlipidemia. It has become apparent that, in children, tofacitinib increases the risk of chickenpox which correlates with the observed increased rates on *Herpes zoster* flares in adults.

There was also discussion of whether broader JAK-inhibitors might interfere with the erythropoietin and growth hormone signaling pathways leading to anemia and growth delays. Dr. Gadina highlighted, however, that in patients with the autoinflammatory Type-1 interferonopathy, CANDLE, treatment with baricitinib resulted in improved disease control and concomitantly patients resumed relatively normal growth. This suggests that better disease and inflammation control might be predominant over the potential effects of the drug on growth hormone signaling. As many cytokine receptors and growth receptors use JAK-STATs for signaling, including the growth hormone receptor, concerns regarding off target effects of JAK inhibition in children remain till more data become available.

The possibility to combine a JAK inhibitor with a biologic was discussed and compared with a combined therapy of a JAK inhibitor with methotrexate or corticosteroids. So far, the data are limited to few anecdotal cases and larger studies are needed to assess the safety of a combination therapy. Finally, at least in the case of baricitinib, the drug half-life is weight based and shorter in children than in adults, and an increase in frequency of administration and in doses may be needed to achieve therapeutic efficacy.

### Is targeting IFNγ-induced pathways likely to be effective in SJIA-LD? / Dr. Fabrizio De Benedetti

Dr. De Benedetti reviewed evidence supporting targeting IFNγ-related pathways in SJIA-LD. A growing body of evidence, albeit indirect, supports the hypothesis that IFNγ may be a pathogenic mediator of SJIA-LD: 1) the vast majority of SJIA-LD patients have a history of MAS, often recurrent [11–13], and IFNγ is implicated as the pivotal cytokine in MAS; 2) in the 12 months preceding onset of the lung disease, patients with SJIA-LD have rising ferritin and levels are higher than those of SJIA patient without lung disease [13]; 3) a prominent IFN-induced signature is present in lung biopsies of SJIA-LD patients with overexpression of genes specifically upregulated by IFNγ [12]; and 4) mice with t-bet CD4 restricted overexpression develop an inflammatory PAP, characterized by a CD4 infiltrate (similar to that present in SJIA-LD lungs) and by a prominent IFN-γ signature [17]. Finally, in these mice abnormal differentiation of tissue macrophages was demonstrated suggesting a shift towards M1 macrophage and subsequent inability to clear surfactant proteins, again pointing to a derangement of macrophage differentiation as a potential mechanism.

Altogether, these observations suggest that therapeutic neutralization of IFN-γ should be considered as a potential therapeutic approach in SJIA-LD. Emapalumab is an anti-IFNγ antibody that has been approved by the FDA for patients with primary hemophagocytic lymphohistiocytosis (HLH). The preliminary results of the ongoing phase II trial of emapalumab in MAS/SJIA showed complete response in all of the 9 patients enrolled, all of whom had previously failed conventional therapies [34].

### New drug discovery: computational approaches to drug repurposing by reversing gene expression in SJIA-LD / Drs. Grant Schulert & Alex Pickering

Alex Pickering from Harvard Medical School described an innovative computational approach to understanding SJIA-LD and also identifying repurposed drugs. This project is the result of a collaboration between the Cincinnati Children’s, Harvard University and the *Systemic JIA Foundation*. It relies on an approach called connectivity mapping on RNA-Seq data. Connectivity mapping uses a gene expression profile from patients to search for drugs and genetic manipulations that have either similar or opposing effects on gene expression. Drugs that oppose the gene expression changes observed in the patients are predicted therapeutics. Genetic manipulations that either oppose or mimic these gene expression changes suggest a role of the manipulated gene in the disease process.

**Fig. 2:**
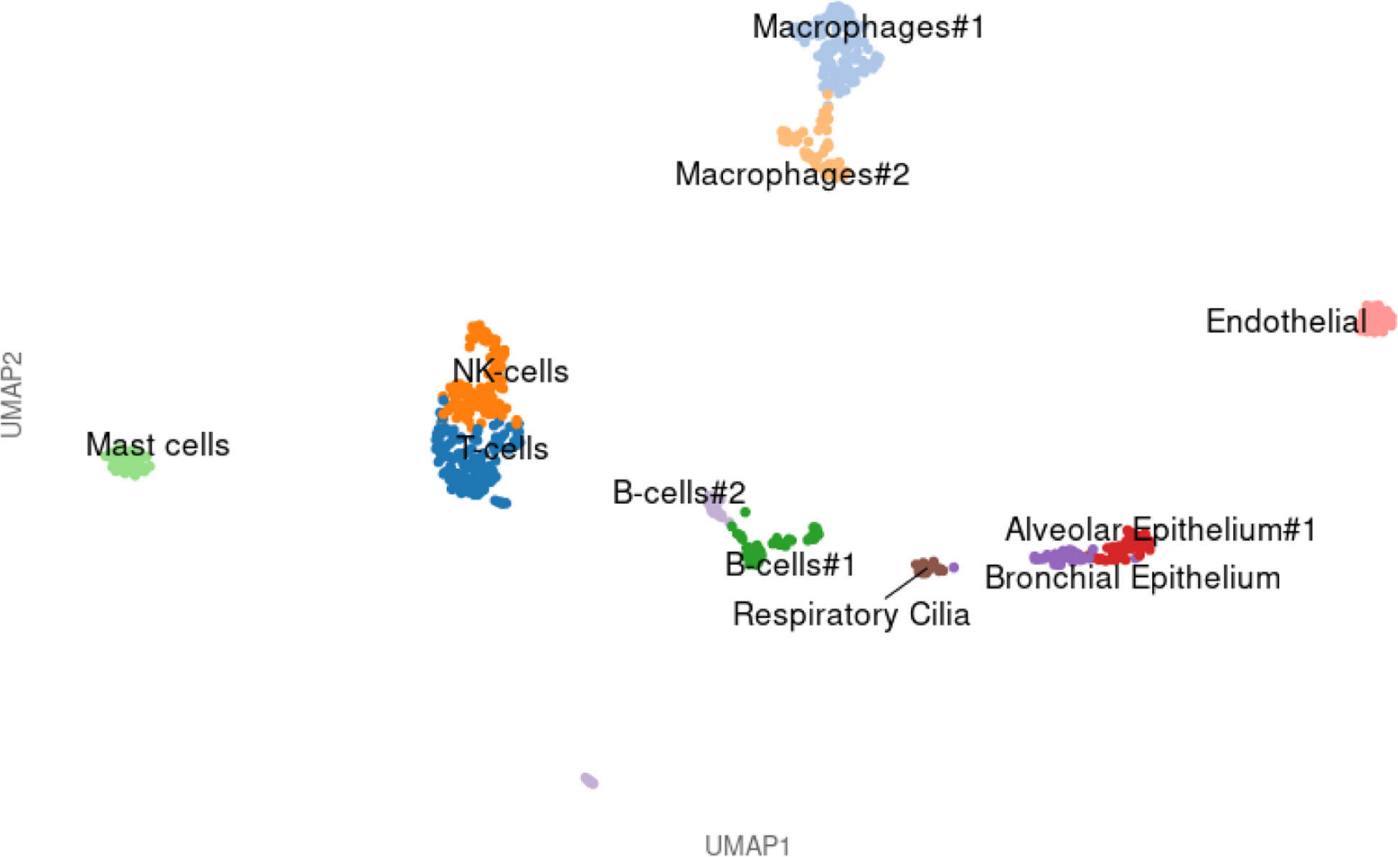
UMAP dimensionality reduction after integrating an affected and unaffected lung sample from a patient with SJIA-LD

**Fig. 3:**
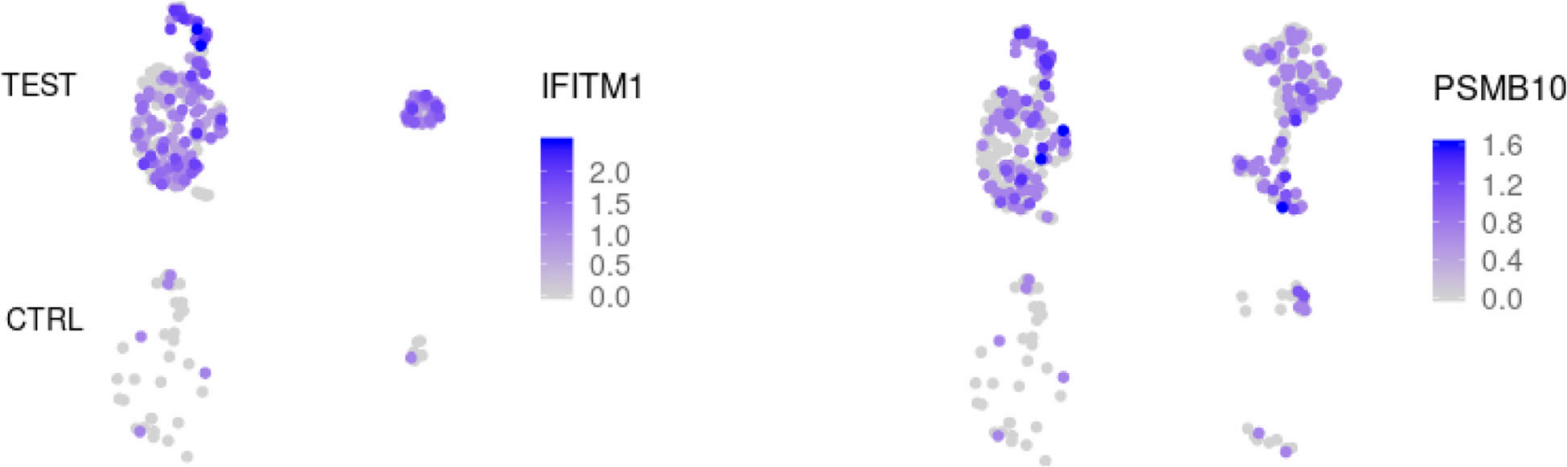
NK- and T-cell clusters split by sample (TEST = affected, CTRL = unaffected) and colored by pre-integration expression of IFITM1 and PSMB10. Analyses performed with Seurat version 3.0

Early results appear promising and highlight the opportunity to identify additional therapeutic targets and approved drugs that can reverse the disease signature. Out of ~ 7000 genetic manipulations, those that correlate most strongly with gene expression in whole lung samples from patients with SJIA-LD either upregulate IFN-γ or NF-kB signaling. IFN-γ signaling in lung NK, T, endothelial, and macrophage cells from a SJIA-LD patient also appeared to be upregulated. As compared to the same cell types from an uninvolved biopsy (Fig. 2), IFITM1, a type I and II interferon induced transmembrane protein that inhibits viral cytoplasmic entry (doi: 10.1074/jbc. M115.657346,) and PSMB10, a IFNγ-regulated component of the immunoproteosome, were among the top most significantly upregulated genes (Fig. 3).

#### Section 5: overlapping clinical phenotypes that may share common disease mechanisms with SJIA-LD

### Patient experience of MAS-liver patients / moderator: Rashmi Sinha; panelists: Zulayka Martinez, Katie Pierce

Patients with lung disease are not the only subgroup of SJIA patients with MAS and predominant organ-specific involvement. Another small, but distinct subgroup of patients from the *SJIA Parents Network* is patients with MAS and liver involvement. Two parents of children diagnosed with SJIA, MAS, and persistent liver issues reported their child’s experience specifically highlighting the connection between recurrent MAS and persistent liver disease.

### Patient story - Zulayka Martinez

Ms. Martinez recounted the story of her daughter, who is now 6 years old. At the age of 4 years, they first started noticing recurrent rashes. Two months later, in April 2018, she started getting fevers as well. The laboratory test pattern pointed to SJIA, but her rashes were atypical, and she had marked generalized enlargement of her lymph nodes. After a long investigative workup which included several MRIs, X-rays, HRCT scans, ultrasound studies, and a bone marrow biopsy, other diseases were ruled out, and she was diagnosed with SJIA. Because of prominent MAS features, the child received multiple pulses of IV corticosteroids and improved. She was also started on anakinra and, initially, had a good response to it. In November 2018, she was unwell again. While general laboratory test results were suggestive of only mild systemic inflammation, the liver function tests were persistently elevated and kept rising (AST/ALT above 200’s unit/L).

In January 2019, she was admitted with rashes and generalized pains. Her medical team decided to replace anakinra with tocilizumab. During the first infusion, she developed a strange pruritic rash, and complained of throat pain that compromised her ability to swallow. She finally improved, mainly in response to IV corticosteroids. Anakinra was added back to the tocilizumab. She responded well to the addition of anakinra and was better for a few days, but then the inflammatory activity started rising again. During the second infusion of toclizumab, she started to gag, and her blood pressure dropped. The infusion was stopped immediately, and the treatment with tocilizumab was permanently discontinued.

By the beginning of February 2019, her liver function tests were rising again (AST 425 unit/L, ALT 512 unit/L), and her serum total IL-18 level reached 364,588 pg/mL (up from 183,292 pg/mL 2 months earlier). By the end of February 2019, she was admitted for increasing liver enzymes (AST 981 unit/L, ALT 704 unit/L) associated with moderate hyperferritinemia (ferritin 465 ng/ml), which could not be controlled even with IV pulse corticosteroids. Hepatology was consulted and a liver biopsy was obtained. The liver biopsy results were inconclusive, but did not show signs characteristic of drug injury. Her team felt comfortable increasing the dose of anakinra, but within 4 days, her liver enzymes further increased (AST - 4070 unit/L, ALT - 4557 unit/L). Her ferritin was 540 ng/ml, and LDH reached 8170 unit/L. Anakinra was discontinued, and her SJIA symptoms returned: rash, fevers, intense body and throat pain.

She got some relief with IV corticosteroids and was next started on canakinumab. Over the next few weeks, her inflammatory markers started improving. The liver markers mildly improved, but still remained highly elevated (LDH 2575 unit/L, AST 1206 unit/L, ALT 2079 unit/L), and total IL18 was at 245,581 pg/mL. Regularly scheduled pulse steroids were used to keep the liver disease under some degree of control. In the meantime, additional staining of the liver biopsy with anti-CD163 and anti-CD8 antibodies revealed histopathologic features suggestive of MAS.

Liver enzymes started rising again and in the fall of 2019, tacrolimus was added to her treatment regimen with some improvement: within a week, LDH dropped from 4020 to 1753 unit/L; AST from 1034 to 241 unit/L, and ALT from 1601 to 499 unit/L.

Ms. Martinez noted that her daughter’s symptoms were primarily fevers, rash, pain, sore throat and elevated liver function tests. She did not have true arthritis. Her team has found serum total IL-18 to be very useful in tracking her disease activity, and it is now being monitored monthly. LDH seems to be another useful marker.

She also noted that the only medication that works for her daughter are IV corticosteroids, and she now receives an infusion at least weekly, and often more frequently when the child is in a flare. She is tired of the many side effects that her child is experiencing including marked growth retardation, weight gain, and mood changes. She would like to have more medication options suitable for cases of SJIA, MAS and associated liver disease.

Since the NextGen conference, Ms. Martinez’s daughter has had a few mild flares. However, since adding tacrolimus, her daughter’s lab test abnormalities have improved and overall she has been doing better. The frequency of canakinumab injections has been increased. Oral corticosteroids are being slowly tapered, but IV corticosteroid pulses are still administered every 2 weeks.

### Patient story - Kate & Jonathan Pierce

Mr. and Mrs. Pierce’s 2 years old daughter was diagnosed with SJIA at the age of 8 months after several weeks of recurrent fevers and rash. She was initially suspected to have atypical Kawasaki disease, but she did not respond to IVIG, and several ECHO studies were normal. After further testing, the diagnosis was changed to Systemic JIA. Her disease was initially controlled with anakinra and prednisone, but after 3 months of the treatment, with corticosteroid taper, her fevers and rash returned. In spite of switching biologics to canakinumab and increasing her prednisone dose, her fevers persisted and inflammatory markers continued to rise.

In October 2018, she developed MAS. She recovered quickly with tocilizumab and IV corticosteroids, but 10 days later, the MAS recurred for a second time. This MAS episode was more difficult to treat and was characterized by increasing liver inflammation in spite of improvement in all other MAS markers. During this hospitalization she was found to have cytomegalovirus (CMV) infection which was thought to explain her persistent liver inflammation. The inflammation was eventually controlled and her steroids were slowly tapered.

She received five infusions of tocilizumab, before she developed an anaphylactic type reaction to the drug. After this reaction, tocilizumab was replaced with canakinumab, and the dose of corticosteroids was increased again followed by a very slow taper.

Mrs. Pierce noted that her daughter’s typical SJIA symptoms were fever, rash, and enlarged spleen and liver. She did not have true arthritis. Remarkably, her total IL-18 levels were always very elevated, particularly during or right after MAS. After the last MAS episode, total her IL-18 was at 361,000 pg/mL.

By the beginning of 2019, once she completed her steroid taper, her LFTs were again elevated, though the rest of lab test results were mostly normal (ferritin in the 30s ng/mL, still low hemoglobin, with WBC count of 8.0 K, and platelet count at 200 K). CMV was no longer detectable in her blood.

A liver biopsy was performed in April 2019. Though results of the biopsy were inconclusive, there were “punched out lesions of hepatocellular necrosis” which were felt to be related to the inflammation from SJIA or MAS. There were no signs of active CMV infection in the liver.

For the past year, she has struggled with persistent inflammation causing nightly fevers and elevated inflammatory markers, including liver enzymes. She has been on corticosteroids for most of this time, causing significant growth delay.

Since this summer, her team has been trying different drug combinations to effectively control her inflammation. They first tried adding anakinra to the canakinumab for a month. When that did not help, they next tried adding tacrolimus to her regimen. For about a month, her labs completely normalized, but then the inflammatory markers started rising again and fevers returned.

Her team did a renewed search for drugs to control her inflammation, and considered a range of options including Rilonacept, thalidomide and JAK-inhibitors. She was recently started on tofacitinib with the hope that this will result in better control of her inflammation and help wean corticosteroids. However, her daughter has been flaring again recently. Her team is continuing with tofacitinib, but also started the initial evaluation for possible hematopoietic stem cell transplantation.

Mr. and Mrs. Pierce are interested in other drug options to control her daughter’s inflammation, as steroids have severe side effects when used long term, and none of the biologic medications can control her child’s disease, especially the MAS and liver inflammation.

### Pathology in recurrent MAS with liver involvement / Drs. Alexei Grom & Fabrizio DeBenedetti

It has been recognized that a subset of patients who do not meet full criteria for SJIA or overt MAS, may have recurrent or “smoldering/incomplete MAS”. A proportion of these patients have predominantly liver involvement. Wouters, et al. previously described typical histopathologic findings in these patients: sinusoidal inflammatory infiltrate is a prominent feature and consists both of increased number of CD8+ T lymphocytes and highly activated Kupffer cells with some of them exhibiting hemophagocytic activity [35]. The CD8 immunostaining in these patients typically identifies large numbers of CD8 positive lymphocytes both in the portal infiltrates and in the sinusoidal infiltrates. The CD163 immunostaining marks large numbers of activated macrophages with plump contours both in the portal areas and in the sinusoids. Immunostaining for cytokines identifies CD8+ T cells as the main producers of large amounts of IFN-γ.

Consistent with this study, three patients with predominant liver involvement recently evaluated by Dr. De Benedetti (2 of whom fully met the ILAR criteria for SJIA), had similar histopathologic pattern, including large numbers of CD68+ macrophages. Their liver biopsies showed highly increased levels of mRNA for IFNγ-induced genes, while other classical pro-inflammatory cytokines were not markedly increased, including IL-18, IL-6 and TNF-α. Type I IFN induced gene expression was also roughly normal. These observations suggest that IFN-γ and IFN-induced pathways might be an attractive therapeutic target in this patient population as well [36].

### “Persistent partial MAS” as the common link between SJIA-LD and MAS-liver patients / group discussion

The discussion was then focused on “persistent partial” or “smoldering”” MAS as the common link between both sets of patients.

### Parent observations

Several parents of both SJIA-LD and SJIA-Liver patients described that their children were in a state of what was best described as “smoldering MAS” for prolonged periods of time. They would have low-grade fever, some rash, and fatigue. Lab tests would show some features of MAS, but not full-blown MAS. Clinicians who had treated such patients agreed with this observation and emphasized that treating such patients with existing medications was challenging.

Since most children with SJIA-LD have a low level of persistent inflammatory activity and some laboratory abnormalities typical of MAS without meeting the full Ravelli’s 2016 MAS criteria [37], the point was made that a substantial proportion of children with SJIA-LD might have “smoldering” MAS. The concept of “smoldering” MAS has not been formalized, and since this is a potential target for any future studies and clinical trials, the group sought to generate a preliminary definition, criteria and possible an alternative term to describe this state. The group discussed several terms for this clinical state, and while no consensus was achieved, a majority of participants endorsed the phrase “persistent partial MAS”.

The group identified the potential characteristics to define “persistent partial MAS”: in a patient with active SJIA and persistent inflammation (provided that infection or other causes have been ruled out), there should be newly worsening or persistently abnormal values for at least 6 weeks (with inability to taper medications because of worsening values) indicating:

- liver abnormalities
- disorder of hematopoiesis
- coagulopathy
- highly elevated serum IL-18 with modestly elevated CXCL9

The presence of all 4 of these should support the diagnosis of “persistent partial MAS”. Numeric laboratory values for the three components of the definition still needs to be defined in the future.

#### Section 6: planning for future clinical trials

### Alternative designs for a trial for recurrent MAS with organ (lung / liver) involvement

Dr. Rashmi Sinha then summarized the patient perspective regarding the need for new treatments for patients with MAS and lung / liver involvement. The entire group recognized that patients who have recurrent MAS associated with SJIA, SJIA-LD or MAS with Liver Disease not only have high mortality, but also have disease- and medication-related morbidities that slowly accumulate, affecting all aspects of the child’s health-related quality of life. With input from representatives from the FDA, possible approaches to the design and implementation of a clinical trial for this population were discussed. Potential therapeutic strategies included neutralization of IFN-γ with emapalumab, JAK-inhibitors, and/or IL-18 inhibitors.

The group felt that the study design should address the current unmet need, take into consideration patient reported outcomes, and should also meet the regulatory requirements to gain approval for this indication. Appropriate outcome measures should be considered including patient reported outcome (PRO) measures as well as meeting (as yet unidentified) “MAS remission” criteria. There was an agreement that the ability to taper corticosteroids, as well as other concomitant medications, should be avoided as a primary outcome measure, but must be used as secondary measures, given the clinical relevance of the comorbidities associated with the multiple chronic therapies of these children.

### Developing patient-reported outcomes measures for clinical / session moderated by: Dr. Hermine Brunner

Dr. Brunner reviewed currently available PRO measures of health-related quality of life that have been validated and including those used in the clinical trial of tocilizumab in SJIA [6]. These PRO measures, however, were mainly aimed at the assessment of the effects of the arthritic and, to a lesser degree, the systemic components of the disease. Therefore, the current PRO measures are unlikely to be sensitive enough to fully capture “persistent-partial” SJIA and/or SJIA-LD, and future work is needed to develop such a measurement tool.

### Summary of parent observations

Parents shared what signs they use to judge whether their child is doing better or worse. They described both systemic and lung specific symptoms, but also reported a complex interaction between the lung and systemic symptoms of the underlying SJIA (e.g., a child might be affected due to shortness of breath (due to lung symptoms), and/or tired and achy (due to systemic symptoms). Potential outcome measures suggested by the parents are listed in Table 2.

**Table 2:** Potential Parent Reported Outcome Measures - For Systemic & Lung Disease

Lack of energy / decreased physical activity/being sluggish	
Behaviors like sitting on the couch and playing with iPad, or just sitting quietly	
Fatigue - reporting tiredness after minor exertion	
Disturbed sleep at night	
Not wanting to get out of bed	
Grumpy behavior: being annoyed without reason, with siblings and parents	
Less smiling and laughing behavior	
Reduced exploratory behavior / reduced sense of curiosity	
Decreased interest in play	
Lack of appetite	
Cough	
Labored breathing	
Breathlessness with every day-life activities (i.e., climbing stairs)	
Decreased ability to run around	
Increased respiratory rate	
O2 requirement (for patients who use oxygen supplementation)	

The parents then discussed potential “lung-relevant” PRO candidate items that, from their prospective, could distinguish between worsening lung disease and worsening systemic component of SJIA (Table 3).

**Table 3:** Lung-relevant PRO Candidate Items

	Lung Disease - Specific	SJIA - Specific
**Symptoms**	Flushing	Fever
	Cough	Rash
	Labored breathing	Chest pain
	Pruritis	Joint pain
		Joints stiffness
	Fatigue	
	Low energy	
	Decreased appetite	
	Decreased physical activity	
	Feeling sad	
	Lack of engagement/participation	
	Lack of curiosity	
	Decreased interest in play	
**Signs**	Heart rate	Fever
	Respiratory rate	Rash
	Oxygen requirement	Joint swelling
	Growth failure	Hepatomegaly
	Erythematous clubbing	Splenomegaly
		Lymphadenopathy
		Poor linear growth

For further refinement, the list generated will be (1) disseminated among a larger group of parents through the *Systemic JIA Foundation* network, and (2) discussed with experts to explore whether the Patient-Reported Outcomes Measurement Information System (PROMIS; www.healthmeasures.net) could be used to design a PRO measure to be used in trials of medications to treat SJIA-LD.

### Measuring and tracking lung disease progression: options & challenges, Dr. Grant Schulert

Dr. Schulert reported on his work with Dr. Yukiko Kimura to establish validated criteria to measure and track lung progression through the CARRA Registry network. He presented and discussed components of this measure including a lung specific global assessment (SJIA-LD Severity Score), and physician lung global assessment (PLGA). Potential specific items could include oxygen requirement, cough, exercise intolerance, therapy intensity, and the degree of the redness of the digits as well as the % of the involved area of the lungs on HRCT. Drs. Schulert and Kimura hope to establish a cohort through the CARRA Registry to collect a small number of such measures prospectively to track disease evolution, along with obtaining biosamples through the CARRA Biobanks in the US and Canada.

#### Section 7: conclusions & future steps

### Plans for a new international refractory SJIA project: ReSyst

The meeting recognized that there are subgroups of refractory SJIA patients (including SJIA-LD, liver-predominant MAS, and resistant/destructive arthritis, among others) who need to be identified quickly and treated appropriately. Identifying the appropriate treatments will mean addressing the challenges of 1) small patient numbers, 2) high patient complexity/severity, and 3) the lack of approved treatment options. While the rest of the meeting focused on the efforts to understand and treat SJIA-LD, there is also a clear need for a parallel effort to recognize and understand refractory SJIA in general.

The meeting closed with a discussion between clinicians, researchers, and parents regarding the efforts to create an international consortium focused on understanding of treatment-resistant SJIA (ReSyst). This effort includes groups from the US, Canada, the Netherlands, and Italy. Such an effort would leverage several existing networks including Pediatric Rheumatology European Society (PReS), the European Reference Centers, Childhood Arthritis Research Alliance (CARRA), and UCAN CAN-DU. As a first step, the group agreed upon a provisional definition of refractory SJIA shown in Table 4.

**Table 4:** Provisional Classification Criteria for Refractory SJIA

Failure to respond to IL-1 AND IL-6 blocking biologics (failure = inability to resolve arthritis, systemic symptoms, or liver dysfunction, or being steroid dependent) OR ≥2 episodes of MAS in a 2 year period OR Development of SJIA-LD	

More discussions will follow regarding funding and the logistics of collaborations needed between the organizations to collect clinical data and biospecimens on these patients.

## Conclusions

In conclusion, the mortality rate of children with SJIA-LD is high, and effective targeted treatments remain elusive. This underscores the urgent need to diagnose SJIA-LD early, elucidate risk factors, and develop therapeutic strategies to improve its prognosis. Environmental and genetic factors are likely to contribute to the development of the lung disease, but increasing evidence suggests that SJIA-LD may be caused by alveolar macrophage dysfunction with contributions from the SJIA/MAS inflammatory milieu and possibly, from increasing use of the IL-1 and IL-6 blocking therapies. Potential novel therapeutic strategies include targeting (1) IFN-γ with the monoclonal anti-IFNγ antibody emapalumab; (2) the IFNγ-induced pathways with Janus kinase inhibitors, and (3) IL-18 with the IL18-blocking agent tadekinig alfa or other agents. Any potential clinical trial design in Refractory SJIA, or SJIA-LD more specifically, should address the current unmet need, should take into consideration patient reported outcomes, and should aim for regulatory approval for this indication. Importantly, the perspective of patients and families must be frequently and seriously considered at all phases of such trials.

## Data Availability

All the data discussed during the meeting have now been published and appropriately referenced at the end of the manuscript.

## Abbreviations

ACR: American College of Rheumatology
AOSD: Adult onset still’s disease
APOE: Aapolipoprotein E
BAL: Bronchoalveolar lavage
CARRA: Childhood Arthritis Research Alliance
CMV: Cytomegalovirus
ECMO: Extra-Corporeal membrane oxygenation
sHLH: Secondary hemophagocytic lymphohistiocytosis
HRCT: High-resolution chest computer tomography
ICU: Intensive Care Unit
ILD: Interstitial lung disease
IFN-γ: Interferon gamma
IV: Intravenous
IVIG: Intravenous Immunoglobulins
LFT: Liver function test
Mo-AMs: Monocyte-derived alveolar macrophages
MRI: Magnetic resonance imaging
NIAID: National Institute of Allergy and Infectious Diseases
NIAMS: National Institute of Arthritis and Musculoskeletal and Skin Diseases
PAP: Pulmonary alveolar proteinosis
PRO: Patient reported outcome
PReS: Pediatric Rheumatology European Society
scRNA-Seq: Single-cell RNA-sequencing
TR-AMs: Tissue-resident alveolar macrophages
WBC: White Blood Cells
